# Pathological and Evolutionary Implications of Retroviruses as Mobile Genetic Elements

**DOI:** 10.3390/genes4040573

**Published:** 2013-10-24

**Authors:** Madeline Hayes, Mackenzie Whitesell, Mark A. Brown

**Affiliations:** 1 Department of Biology, Colorado State University, 801 Oval Drive, Fort Collins, CO 80523, USA; E-Mail: hayesmad@rams.colostate.edu; 2 Department of Environmental and Radiological Health Sciences, Colorado State University, 801 Oval Drive, Fort Collins, CO 80523, USA; E-Mail: mwhitese@rams.colostate.edu; 3 Department of Clinical Sciences, Colorado State University, Colorado State University, 801 Oval Drive, Fort Collins, CO 80523, USA

**Keywords:** transposon, mobile genetic element, virology

## Abstract

Retroviruses, a form of mobile genetic elements, have important roles in disease and primate evolution. Exogenous retroviruses, such as human immunodeficiency virus (HIV), have significant pathological implications that have created a massive public health challenge in recent years. Endogenous retroviruses (ERVs), which are the primary focus of this review, can also be pathogenic, as well as being beneficial to a host in some cases. Furthermore, retroviruses may have played a key role in primate evolution that resulted in the incorporation of these elements into the human genome. Retroviruses are mobile genetic elements that have important roles in disease and primate evolution. We will further discuss the pathogenic potential of retroviruses, including their role in cancer biology, and will briefly summarize their evolutionary implications.

## 1. Introduction

In order to gain a better understanding of the impact of retroviruses on human health, it will be important to focus research efforts on both the pathology of retroviruses and their role in the evolution of the human genome. A complete understanding of retroviruses must include knowledge of their relationship to other mobile genetics and transposable elements specifically.

Transposable elements are mobile genetic elements that have been defined as sequences within DNA that are repetitious and have the ability to relocate within a genome or be integrated into a new genome [1,2]. The prevalence of transposable elements within a genome varies by species and it has been reported that between 40%–45% of the human genome is comprised of these elements [2,3]. There are two classes of transposable elements that are commonly addressed in the literature. Class I includes retroelements that utilize an RNA intermediate during replication and transpose during DNA transcription [2,4,5]. Class II do not require an RNA intermediate as they transpose during DNA synthesis [2,4,5]. More recently, eukaryotic transposable elements have been classified simply as retrotransposons and DNA transposons [6].

Another type of transposable element includes miniature inverted repeats (MITEs), which is a class of short elements whose mechanism of transposition is not yet entirely understood and which share traits of both class I and class II elements [4]. MITEs are less than 600 base pairs in length and lack an open reading frame [7,8]. 

Class I elements, or retroelements, encompass interspersed nuclear elements (both long and short) and long terminal repeat (LTR) retrotransposons, which are comparable to integrated retroviruses [4,5]. Long Interspersed Nuclear Elements (LINEs), which are the most prevalent type of autonomous retrotransposons in humans, are characterized as being several kilobases long and comprise 17%–18% of the human genome [9,10,11]. LINEs lack LTRs and typically code for the enzymes endonuclease and reverse transcriptase [9,12]. One type of LINE elements, called L1 elements, are the only form of autonomous transposable elements that are active in humans today [12]. A small portion of L1 elements can be pathogenic or beneficial to the host, depending on where they insert in the host genome [9,10,12]. Short Interspersed Nuclear Elements, or SINEs, are nonautonomous, as they do not code for protein synthesis [10,12]. Thus, in order to become mobile, SINEs require the proteins that are coded for by LINE elements [10,12]. Alu elements are SINEs that occupy approximately 5% of the human genome [13].

## 2. Retroviruses

### 2.1. Retroviral Structure and Origin

Many class I transposable elements share important characteristics with retroviruses [14]. The basic structure of retroviruses is relatively homologous, as they all use a DNA intermediate for replication and all have LTRs [15]. The coding region of retroviruses can be divided into two domains. The first is the gag-pol domain. The gag region codes for nucleocapsid and matrix proteins, and the pol region for the viral protease, reverse transcriptase and integrase enzymes [15,16]. The second domain, *env*, codes for surface proteins that bind to host cells [15,16]. The coding regions are flanked by LTRs. The LTRs contain the promoter sequences, enhancers, repressors, and a poly-adenosine (poly-A) tail [17]. It is important to note that, while this characterization of the basic structure of retroviruses applies to all variations, many retroviruses have additional genes that are important for their function. 

Sequences similar to reverse transcriptase have been discovered in both retroviruses and transposable retroelements with and without LTRs [14]. Howard Temin, after his discovery of reverse transcriptase in 1970 and the protovirus hypothesis of 1971, suggested that retroviruses originated from transposable elements [18,19,20]. Since then, literature has accumulated which suggests that retroviruses and transposable elements have a common ancestor [21,22]. Still, retroviruses and retrotransposons are distinguished from each other, most notably by the *env* gene present in retroviruses that enables them to create proteins for viral envelopes [16,22].

### 2.2. Endogenous Retroviruses and Pathogenic Potentials

In addition to their similarity to transposable elements, endogenous retroviruses (ERVs), which are retroviruses incorporated into the germline, show many similarities to exogenous retroviruses [17,23]. This suggests that ERVs may have origins in pathogenic exogenous retroviruses [17,23].

Retroviruses are unique among animal viruses in that their replication is completely dependent on their integration into the DNA of the host [15]. This provides an important target for the immune system and retroviral drugs in attempting to inhibit retroviral action. In order to inhibit integration, some pharmaceuticals seek to interfere with reverse transcription, which is the mechanism of azidothymadine (AZT) used in the treatment of human immunodeficiency virus (HIV) [24]. Cells of the immune system may also contain genes that have a role in the direct prevention of viral replication through targeting the growth of infected cells [23]. Furthermore, as is true with any virus, the action of retroviruses depends on their ability to effectively evade the host’s immune system, and make entry into host cells via surface receptor proteins [23]. Consequently, if the surface proteins of the cell are incompatible with those of the virus, receptor-binding inhibition is achieved [23].

If a virus is able to evade the immune system and incorporate into the genome it is dubbed an endogenous retrovirus (ERV), and can have a myriad of impacts. These fall into two broad categories: parasitic and symbiotic (or functional) [25]. The parasitic theory states that ERVs (and some other repetitive genomic elements) use the host to replicate while offering no benefit to it [26]. These retroviruses are therefore pathogenic, as they can cause disease through inhibition of the cell cycle of host cells.

Conversely, the symbiotic, or functional, theory proposes that endogenous retroviruses are conserved in the genome because they offer a benefit to the host or have a functional role in host processes [25,27]. Specifically, there is evidence that some ERVs can hinder exogenous retroviruses, thus providing a level of resistance in the host [17,23]. Despite their opposing propositions, the parasitic and symbiotic theories are not entirely contradictory, as the function of the ERV differs depending on its specific characteristics and the location in which it is inserted in the host genome [23].

In its most basic form, insertion of a genetic element can be problematic for the genome by being inserted in a coding region. If an element interrupts a gene, it is possible that the gene will lose function, as is true with any insertion or deletion mutation. If the gene does not lose function, the promoter sequences found in the LTRs may increase expression of downstream genes [16]. LTRs can be found throughout host genomes, left behind when a transposable element replicates and transposes and may have significant impacts on gene expression (detailed below) [16]. However, once an ERV incorporates into the host genome, it becomes vulnerable to mutation within that genome [28]. Consequently, most observed ERVs have lost function [28,29].

### 2.3. Effects of Reactivation

Even after being rendered non-functional, ERVs can regain functionality through recombination or environmental factors [22]. Recombination will promote diversity and may occur with other endogenous sequences, or active exogenous viruses. If LTRs of two elements recombine, they may form a strengthened promoter [17]. If the env regions recombine, they may change the surface proteins of the virus, thereby potentially broadening the range of hosts for the virus [17]. This mode of reactivation is of concern in the use of viral vectors in gene therapy, as recombination may lead to a harmful viral infection [17,30]. It has been of particular concern when assessing the safety of utilizing lentivirus vectors [30].

Once these recombination events take place, environmental factors like ultraviolet (UV) radiation may reactivate the virus, causing it to produce viral particulates [31]. This reactivation by UV radiation has been shown to occur in rodent models with other types of viruses, including HSV-1 [32,33]. In addition, it has been demonstrated that the methylation status of certain retroelements (including L1 and HERV-K) may affect their expression in tumors and, specifically, demethylation may contribute to their ability to transcribe, thus potentially producing an effect within the host [34,35].

### 2.4. Oncological Implications

Reactivation of ERVs is only one of several mechanisms through which transposable elements may negatively affect the genome. Retroviruses (both endogenous and exogenous) have been shown to play a significant role in cancer development, including breast and colon cancers [36,37,38,39] .Certain viruses, known as acute transforming oncogenic retroviruses, cause disease via the oncogenes they carry (most likely gained form cellular oncogenes) [40]. These viral oncogenes, although carried by replication defective viruses, can induce tumors rapidly [40]. Non-acute transforming oncogenic retroviruses contain no such viral oncogenes (and are usually replication competent), but insert viral DNA near a cellular proto-oncogene, and through their own promoter, can cause an over-expression of proto-oncogenes in the cell [40,41].

These observations regarding cancer-causing viruses and reactivation may have important implications for cancer research. For example, one study observed the transformation of adherent melanoma cells to malignancy *in vitro* under stress as a result of the reactivation of Human Endogenous Retrovirus-K (HERV-K) [42]. Furthermore, another study found that UVB radiation activated HERV-K in human melanoma cells, which may be an important step in the mechanism through which UV radiation causes skin malignancy [31]. When considered in conjunction, these findings could hold much significance in early detection and treatment of melanoma [31]. Recent research has also been exploring the exact mechanism through which HERV expression can lead to cancer, though it is clear that more needs to be done in this area [43].

Other transposable elements, such as LINE elements, have also been linked with cancer [11,34,44]. The structural similarities and basic laws that govern the function and expression of these elements, such as activation of LTRs (which contain promoters), are easily connected with the over-expression of oncogenes and interference with tumor suppressors, if inserted into the correct location in the genome [45]. These principles may even compliment the somatic mutation theory (SMT), which dominates proposed causes of cancer today, as insertion of a viral DNA sequence or transposable element is a form of mutation [45]. Specifically, there is recent evidence of L1 insertions in tumors in human patients with lung cancer, possibly indicating a high level of mobilization of L1 in the tumor cells [44]. There is also some evidence that L1 insertions can lead to tumorigenesis through mediation of retrotransposition in hepatocellular carcinomas [11]. Verification of these associations, as well as efforts to investigate L1 insertions in other cancer types, will be an important area of study in future efforts to understand and combat cancer.

### 2.5. HERVs and Evolutionary Implications

The ability and tendency of Human Endogenous Retroviruses to negatively impact the genome calls into question the evolutionary benefit of conservation of such elements. However, in addition to their pathogenic potential, HERVs can positively affect the genome as well, as they may have played a vital role in mammalian evolution [46].

### 2.6. Known Conserved Elements: Symbiotic Genomic Effects

Although the promoters of HERVs have been shown to have negative effects through oncogenes and proto-oncogenes, they may promote the expression of beneficial genes in other areas. There are several known examples of this. The human salivary amylase gene is regulated by an ERV sequence [47,48]. A highly conserved zinc-finger sequence in primates, ZNF80, has been shown to be controlled by the LTR of ERV-9 [49]. The regulatory sequences of other LTRs are related to the regulatory sequences found in cytochrome complexes [50]. Finally, both HERV-F and HERV-K are found in human placental expression [51,52]. In these cases, ERVs have been established as necessary for functioning of the host and have become an integral part of human physiology.

Placental expression is a particularly salient example of HERVs potentially playing a beneficial role in humans. Along with HERV-K and HERV-F, high expression of ERV-3 has been observed in human placental cells [53]. This is thought to have evolved to accommodate the paradox of placental pregnancy: the fetus is extremely vulnerable to attack during development, particularly from the mother’s own immune system [54,55,56,57]. There is evidence that ERV-3 inhibits this attack by acting as an immunosuppressant towards the mother’s immune cells [53,58]. There is also some evidence supporting the role of syncytin-1, a protein coded for by HERV-W in placental cells, in inhibiting maternal immune cell response to a developing fetus [56]. However, some of the literature is contradictory regarding syncytin-1, as another study found that while syncytin-2 (also coded for by an HERV) does have immunosuppressive characteristics in placenta, syncytin-1 does not [58].

The ability to act as an immunosuppressant not only poses a logical explanation for the conservation of several HERVs, but also may provide a mechanism for the mass accumulation of repeated elements. With the high expression of immunosuppressive HERV elements comes an increased chance of infection by other retroviruses. This allows for the observed mass of defective (but unique) repetitive elements in the genome of mammals [57]. However, such a mass does not appear in other vertebrates, as the expression of ERV elements is slightly differentiated from species to species [57].

While HERVs may be instrumental in protecting a fetus during development and consequently likely had a role in the evolution of placental mammals, excessive HERV activity can also lead to autoimmune diseases [16,59,60]. It is clear that further research needs to be done to reconcile conflicting theories and observations about the effects of HERV activity. It is also important to note that, though HERVs have the potential to be either harmful or beneficial, the presence of an HERV can also be insignificant, with no observed ramifications.

### 2.7. Primate Evolution

In addition to their function in human immunity, HERVs and other mobile genetic elements are thought to have played a role in primate evolution. It is known that most HERVs entered the genome around the time of the divergence of Old and New World Monkeys, 30–45 million years ago [29]. However, some have been found to predate this separation, entering the genome up to 60 million years ago [61]. The time periods in which LTRs, and solitary LTRs in particular, were integrated suggest they may have been a byproduct of, or possibly played a role in, key developmental divergences in the primate lineage [61,62]. Specifically, there is some evidence that several HERV elements that are associated with disease entered the genome as little as 1.2 million years ago, which could account for recent phylogenetic divergence [62]. Acknowledging both the pathogenic and beneficial roles of HERVs and other mobile genetic elements will enable a deeper understanding of their role in primate evolution. Further research in this area will be important to enhancing our understanding of the role of HERVs in evolution and in elucidating the phylogeny of recently diverged primate species.

## 3. Future Directions

In providing an overview of the known pathological and evolutionary implications of mobile genetic elements, and retroviruses in particular, this review serves as a basis from which to proceed with future research efforts. It will be important to expand research regarding the role of ERVs in autoimmune diseases, as well as their significance in maternal immune inhibition during fetal development. This may involve exploring the functionality of proteins coded for by HERV-W and ERV-3. Through continued research in these areas, it may be possible to identify key contributing factors to autoimmune disease, as well as spontaneous abortions. The identification of environmental factors that can reactivate HERVs (including HERV-K) will also be vital in future medical research, particularly as it relates to advancing our knowledge of cancer biology. Other transposable elements, including LINEs, also deserve attention regarding their relationship to cancer development.

Studies of reactivation of HERVs will also be crucial in the field of gene therapy. The potential for recombination of HERVs with newly introduced genes to generate pathogenicity warrants continued investigation in order to make gene therapy a safe procedure. This is of particular interest with lentivirus vectors, and the scientific community should proceed with exploring gene therapy side effects.

Furthermore, as evolutionary biologists seek to construct accurate phylogenetic trees, HERV presence will likely serve as a key tool in assessing the molecular clock of primate divergence in the past few million years. If the common lineage of transposable elements and retroviruses is pieced together, significant progress could be made in elucidating the history of evolution and placental development.
